# Dietary Sources of Melamine Exposure among US Children and Adults in the National Health and Nutrition Examination Survey 2003–2004

**DOI:** 10.3390/nu12123844

**Published:** 2020-12-16

**Authors:** Melissa M. Melough, Deborah Foster, Sheela Sathyanarayana

**Affiliations:** 1 Department of Child Health, Behavior, and Development, Seattle Children’s Research Institute, Seattle, WA 98101, USA; sheela.sathyanarayana@seattlechildrens.org; 2 Department of Epidemiology, School of Public Health, University of Washington, Seattle, WA 98195, USA; dfoster4@uw.edu; 3 Department of Pediatrics, University of Washington, Seattle, WA 98105, USA

**Keywords:** melamine, diet, estimated daily intake

## Abstract

Melamine is a high-production-volume chemical and a kidney toxicant. Diet is a key source of melamine exposure, yet little is known about which foods in the US diet may be contaminated. This study evaluated the associations of foods and dietary patterns with melamine exposure using data from 478 US adults and children from the National Health and Nutrition Examination Survey 2003–2004. Melamine concentrations were measured in spot urine samples. Dietary recalls were used to collect dietary data from the day preceding urine collection. Melamine was detectable (>0.09 ng/mL) in 76.2% of the participants’ urine. The geometric mean urinary melamine was 11.563 µg/g of creatinine (standard error (SE): 1.235). In adjusted linear regression models, each additional ounce of processed meats or whole grains was associated with 10.6% (95% confidence interval (CI): 2.7, 19.0; *p* = 0.007) or 17.4% (95% CI: 4.7, 31.7; *p* = 0.006) greater creatinine-adjusted melamine concentrations, respectively. A dietary pattern characterized by high fruit, whole grain, milk, and yogurt intake was positively associated with melamine exposure. In conclusion, processed meats, whole grains, and possibly other plant-based foods may be important melamine sources in the US. Future research should confirm these findings using more recent data and examine the potential health risks of chronic low-level melamine exposure.

## 1. Introduction

Melamine is an organic nitrogenous compound with numerous industrial uses including the manufacture of laminates, dinnerware, adhesives, cleansers, and flame-retardant materials [1,2]. During multiple historical adulteration events, melamine was illegally added to human and animal food sources to enhance apparent protein contents [3,4,5,6]. Melamine ingestion during these events led to the formation of melamine-containing crystals in the renal tubules, resulting in renal injury and numerous fatalities [1,3,4,7]. Recent evidence suggests that beyond its renal effects, melamine may also act as an endocrine disruptor and neurotoxin [8].

Public health agencies have revised guidance on the allowable levels of melamine in animal and human foods in response to these adulteration events [1,9], yet human exposure through environmental contamination remains pervasive [10,11]. Some exposure may occur due to the migration of melamine from food-contacting materials, especially into high acid foods or when products are microwave-heated [1]. More ubiquitous exposure may result from the widespread use of melamine in the chemical industry, leading to the contamination of water, soil, and crops [2,11]. Melamine is also present as a trace contaminant in nitrogen supplements used in animal feeds [12], and is a degradation byproduct of cyromazine, a pesticide and veterinary drug [12]. Animals excrete the majority of ingested melamine, but low concentrations of melamine have been detected in the edible tissues, milk, and eggs of agricultural animals [13,14,15].

Data from China indicate that fruits, beef and mutton, processed meats, and rice are among the greatest dietary contributors of melamine in adults [16]. An analysis conducted in Albany, New York, detected melamine in over 80% of foods analyzed and observed the highest melamine concentrations among dairy, cereal products, and meat [17]. Taken together, these data suggest that low-level contamination of foods is common, and that meat and grain products may be substantial contributors. However, no study has been conducted to comprehensively examine the dietary contributors of melamine in the US diet. Further investigation of the dietary sources of melamine in the US is warranted, as even low-level exposure may impair kidney function in children [18] and adults [19]. The aim of this study was to examine the dietary sources of melamine in the US population. We hypothesized that meat and grain intake would be positively associated with melamine exposure.

## 2. Materials and Methods

### 2.1. Participants and Study Design

This study used data from the National Health and Nutrition Examination Survey (NHANES), a nationally representative survey of US adults and children [20]. We used NHANES 2003–2004, the only survey cycle to date in which melamine data have been reported. A subset of participants aged 6 years and older (*n* = 492) from NHANES 2003–2004 were selected for the measurement of urinary melamine. From this subset, participants missing dietary data (*n* = 13) or with urinary melamine concentrations greater than 5 standard deviations above the mean (*n* = 1) were excluded. In total, 478 participants met these inclusion criteria; participants in the eligible sample were aged 8 years and older.

### 2.2. Dietary Assessment

The dietary intake data were based on 24-h recalls in which the participants reported the foods and beverages consumed during the preceding day from midnight to midnight. The dietary recalls were conducted by trained interviewers using the Automated Multiple Pass Method, a validated five-step approach with integrated memory cues used to obtain complete dietary data [21]. Given the short half-life of melamine [22], we examined dietary intake only from the day preceding the spot urine collection.

### 2.3. Estimation of Food Group Intakes

Estimated nutrient intakes were generated by matching reported intakes to nutrient data from the USDA Food and Nutrient Database for Dietary Studies, 2.0 (FNDDS 2.0) [23]. The MyPyramid Food Guidance System, which was used in the US from 2005 to 2011, translates the Dietary Guidelines for Americans into concrete recommendations for consumers regarding the amounts of foods and beverages to consume from each food group [24]. The MyPyramid Equivalents Database (MPED) 2.0 was developed by translating the foods in the FNDDS 2.0 into 32 MyPyramid food groups. Using the MPED 2.0, we obtained data on the estimated intakes from each food group for each dietary recall.

Our primary analysis examined the associations of meat and grain intakes with melamine exposure. The variables used for this analysis included total meat, red meat, processed meat, poultry, fish and other seafood high in ω-3 fatty acids and low in ω-3 fatty acids, total grains, whole grains, and non-whole grains. Due to the small number of consumers in the sample (*n* < 20), we did not include organ meats in this analysis. In our secondary analysis, we examined the remaining main food groups: dairy, vegetables, fruits, discretionary oils, discretionary solid fats, and added sugars.

### 2.4. Assessment of Dietary Patterns

Because of the intercorrelations among some foods and food groups, attempting to examine their separate effects in statistical modeling can be challenging [25,26]. Therefore, we conducted an exploratory analysis using dietary patterns to characterize overall diets. In SAS v9.4 (Cary, NC, USA), we performed factor analysis using the MPED 2.0 food groups as the underlying variables, which were reduced to four dietary patterns.

### 2.5. Assessment of Melamine and Creatinine Excretion

Urinary melamine concentration was measured in a subset (*n* = 492) of NHANES 2003–2004 participants aged 6 years and older. Melamine was measured in spot urine samples, a method that has been shown to be predictive of 24-h total melamine excretion [22]. Spot urine samples were collected and processed, and then stored frozen until the time of analysis. Briefly, 1 mL urine samples were spiked with 20 µL of an internal standard, diluted, and then purified on a solid-phase extraction cartridge. The cartridges were rinsed to remove contaminants from the urine matrix, and analytes were eluted from the column with 2 mL of 1% formic acid in methanol. The eluent was evaporated to dryness under nitrogen, and the residue was reconstituted with 100 µL of 1% formic acid. Melamine concentrations were measured using high-performance liquid chromatography–tandem mass spectrometry (HPLC-MS/MS) with a limit of detection (LOD) of 0.09 ng/mL [27]. The laboratory and method were certified according to the Clinical Laboratory Improvement Amendment guidelines with appropriate quality assurance and control procedures [27]. Urinary creatinine concentrations were measured in the spot urine samples using the Jaffé rate reaction, in which creatinine reacts with picrate in an alkaline solution to form a red creatinine–picrate complex, which was measured with a Beckman Synchron CX3 analyzer (Beckman Coulter Inc., Brea, CA, USA) [28]. The urinary melamine concentrations were corrected for creatinine concentration to account for variable dilution among the samples [29].

### 2.6. Calculation of Estimated Daily Intake (EDI)

The EDI of melamine for each participant was estimated using the following equation adapted from Shi et al. [16]: EDI = UE × CE × (1/f) × (1/1000). UE was the creatinine-adjusted urinary melamine (in µg/g). CE was the daily body-weight-adjusted creatinine excretion rate. Among children, the CE (in units of mg/kg/day) was set to 18.10 for girls aged 6–8 years, 19.46 for boys aged 6–8 years, 19.34 for girls aged 9–13 years, 20.59 for boys aged 9–13 years, 20.59 for girls aged 14–18 years, and 22.74 for boys aged 14–18 years, based on data from healthy children in Germany [30]. The CE was set to 21.83 and 17.08 mg/kg/day for adult males and females, respectively, based on data from Switzerland [31]. F was the fraction of melamine excreted in the urine relative to the total exposure. Although the excretion fraction has not been directly observed in humans, it is inferred that melamine is excreted in human urine without being metabolized, much like what has been observed in animal studies [1]. Therefore, F was set to 90% based on data from a rodent model [16,32]. The final term in the equation allows for the presentation of the calculated EDI in the units of µg/kg body weight/day.

### 2.7. Assessment of Sociodemographic and Lifestyle Characteristics

Participants 16 years of age and older self-reported demographic variables, including age, sex, self-identified race/ethnicity, and income. A proxy respondent provided this information for participants younger than 16 or those who were unable to answer the questions themselves. The family poverty income ratio (PIR) is the ratio of the reported family income to the poverty threshold, calculated based on the Department of Health and Human Services poverty guidelines and accounting for family size, year, and state [33]. Body mass indices (BMIs) were calculated from measured heights and weights. In children younger than 20 years of age, the BMI statuses were determined using BMI percentiles for age based on the 2000 Centers for Disease Control and Prevention (CDC) Growth Chart [34]. The BMI statuses among adults aged 20 years and older were determined using CDC classifications [35].

Serum samples were collected, processed, and stored according to the NHANES Procedures Manual [36]. In participants aged 3 years and older, serum cotinine was measured using HPLC-MS/MS [37]. The serum cotinine concentrations, which reflect recent exposure to tobacco smoke [38,39], were used to define participants’ smoking statuses as follows: cotinine values of <0.5 ng/mL were defined as nonsmoker, those ≥0.5 and ≤10 ng/mL were defined as exposed to environmental tobacco smoke (ETS), and those >10 ng/mL were defined as active smoker.

Physical activity was assessed in NHANES 2003–2004 using the ActiGraph AM-7164 (ActiGraph LLC, Fort Walton Beach, FL, USA), a physical activity monitor (PAM) that collects objective information on the intensity and duration of locomotion activities such as walking and jogging [40]. Participants were asked to wear the PAM during all activities other than swimming or bathing, as the device was not waterproof. Using programs developed by the National Cancer Institute Division of Cancer Control and Population Sciences [38], the raw PAM data were edited in SAS to exclude invalid or unreliable data, and to summarize valid data as the average daily time spent in moderate or vigorous activity bouts. These data were then used to categorize physical activity levels as inactive (≤15 min/d), slightly active (>15 and ≤30 min/d), active (>30 and ≤60 min/d), and highly active (>60 min/d).

### 2.8. Statistical Analyses

The urinary melamine concentrations were below the LOD in 114 participants (23.8%). Observations below the LOD were imputed by NHANES as LOD/square root of 2. Because this single imputation method may impair estimates of variance and covariance [41], we replaced these singly imputed values with multiple imputation (MI) using the SAS MI procedure. We specified an interval of imputation from 0 to the LOD, 0.09 ng/mL. The missingness of each covariate was less than 5%, except for physical activity, which was missing among 37.7% of the eligible participants. Covariate data were assumed to be missing at random. We employed MI using fully conditional specification, a validated method ideal for large epidemiologic datasets consisting of variables with complex relationships [42]. Imputed datasets (*n* = 10) were used to generate descriptive statistics including the mean and standard error (SE), and regression models in each imputation were combined using Rubin’s rules [43] to generate inferential statistics. All analyses used SAS Survey procedures with weights assigned in NHANES to account for the complex survey design.

Multiple linear regression modeling was used to examine the relationships between the dietary intakes of food groups and dietary patterns with melamine exposure. The outcome variable was the log-transformed creatinine-adjusted urinary melamine concentration. The model covariates were chosen a priori based on scientific evidence [16,44]. Minimally adjusted models included the urinary creatinine concentration (continuous), sex (male, female), and age (continuous). Fully adjusted models additionally included the BMI status (underweight, healthy weight, overweight, or obese), family PIR (continuous), race/ethnicity (Mexican American, other Hispanic, non-Hispanic White, non-Hispanic Black, or other including multiracial), physical activity level (inactive, slightly active, active, or highly active), and smoking status (nonsmoker, ETS-exposed, or active smoker). Because this exploratory study involved the use of multiple tests, we used a Bonferroni correction to reduce the probability of type I error [30]. We conducted 9 tests of different food groups and subgroups in the primary analysis (meats and grains) and 6 tests in the secondary analysis (other main food groups); we compared the regression coefficient p-values in these tests to Bonferroni-corrected thresholds of 0.05/9 = 0.006 and 0.05/6 = 0.008, respectively.

## 3. Results

The geometric mean melamine concentration was 11.563 µg/g of creatinine (SE: 1.235) (Table 1). The geometric mean EDI was 0.251 µg/kg of body weight (SE: 0.026). Females tended to have higher mean creatinine-adjusted melamine concentrations than men (*p* < 0.05), but men and women had similar EDIs. Both the EDI and creatinine-adjusted melamine appeared to be higher in normal and overweight individuals compared to those with either lower or higher BMIs. There were also differences in the creatinine-adjusted melamine concentrations between individuals with varying physical activity levels.

In the multivariable regression analyses, the total intake of meat, poultry, and fish was not significantly associated with melamine exposure (Table 2). Among the various types of meat products examined, processed meat appeared to be most strongly associated with melamine exposure. In the fully adjusted model, each additional ounce of processed meat was associated with 10.6% (95% CI: 2.7, 19.0; *p* = 0.007) greater creatinine-adjusted melamine concentrations. The intake of total grains was not associated with melamine exposure, but each additional ounce of whole grains was associated with a 17.4% (95% CI: 4.7, 31.7; *p* = 0.006) greater creatinine-adjusted melamine concentration in the fully adjusted model.

In the secondary analysis examining other major food groups, each additional cup of fruits was associated with 11.8% (95% CI: 1.4, 23.3; *p* = 0.026) greater creatinine-adjusted melamine concentrations in the minimally adjusted model. However, this association was diminished in the fully adjusted model. None of the other major food groups were found to be associated with melamine exposure.

Using factor analysis, we reduced the food group variables down to four factors, or dietary patterns, observable in our population (Table 3). The first was similar to a classical Western pattern [45,46], characterized by high intakes of solid fats, oils, non-whole grains, added sugars, and cheese. The second pattern was consistent with the Prudent pattern [25], and was rich in a variety of vegetables, nuts and seeds, fruits, fish, and whole grains. The third pattern was similar to the previously described American-Healthy pattern [46]. This pattern was characterized by high intakes of fruit, whole grains, soy, milk, and yogurt, and low intakes of alcohol, potatoes, red meat, added sugars, and oils. The fourth pattern was classified as a Convenience pattern [46] and was rich in foods commonly associated with fast food or ready-to-eat items such as potatoes, added sugars, oils, and processed meats.

Associations between dietary patterns and melamine exposure were evaluated in multivariable regression models (Table 4). The American-Healthy diet pattern was positively associated with creatinine-adjusted melamine concentrations in both minimally and fully adjusted models. The other dietary patterns were not found to be associated with melamine exposure.

## 4. Discussion

In this study, processed meats and whole grains were identified as possible dietary sources of melamine exposure in our primary analysis. We also found that an American-Healthy dietary pattern, rich in fruits and whole grains, was associated with melamine exposure. This result may have been driven in part by the high fruit content of this dietary pattern, as fruits were associated with melamine exposure in our minimally adjusted model. Together, these findings highlight processed meats, whole grains, and fruits as potential sources of melamine exposure in the US diet.

The geometric mean melamine excretion among US adults and children was 11.563 µg/g of creatinine (SE: 1.235), nearly four times greater than the geometric mean of 2.977 µg/g reported in a study of adults in Shanghai [16]. Our observed arithmetic mean of 44.68 µg/g of creatinine (SE: 4.72) was approximately seven times greater than the mean of 6.19 µg/g (standard deviation, SD: 7.96) observed in a sample of 105 adults in Taiwan [47]. These differences may be related to the differing food landscapes between countries and changes in melamine production and use between the 2003–2004 NHANES cycle and 2012, when data from Shanghai and Taiwan were collected [16,47]. This NHANES analysis also included children as young as 8 years old, providing a wider age range than was studied in Shanghai or Taiwan. However, we were surprised to find that melamine exposure was higher in older compared to younger individuals. Among a convenience sample of US children aged 4 months to 8 years of age, we previously observed a mean urinary melamine concentration of 27.4 ng/mL (SD: 141.9) [18], markedly higher than the arithmetic mean of 4.1 ng/mL (SE: 0.4) observed among those aged 8 years and older in NHANES. Children are generally more susceptible to environmental exposures compared to adults because of differing metabolisms [48], higher food and water consumption per unit of body weight [49], and increased hand-to-mouth ingestion [48]. This study could not evaluate melamine exposure levels or sources in young children, but this should be pursued in future work.

Debate exists over the levels of melamine exposure that may constitute a health risk to humans [1]. Using a relatively conservative tolerable daily intake (TDI) value of 3.15 µg/kg/day proposed by Choi et al. [50], the mean EDI in the US is approximately 7.9% of the TDI. Using the more liberal TDI of 63 µg/kg/day set by the US Food and Drug Administration [1], the mean EDI in the US is about 0.4% of the TDI. The established TDI values are based on the clinically significant nephrotoxicity that occurs with high-dose exposures. However, another study using NHANES 2003–2004 data found that melamine exposure was inversely associated with the estimated glomerular filtration rate in adults [51], and a case-control study in Taiwan suggested that low levels of melamine exposure increased the risk of kidney stone formation in adults [19]. Melamine may also affect other organ systems, potentially causing sperm abnormalities and DNA damage [52] and affecting hippocampal synaptic plasticity and behavior in rats [53]. Although our data suggest that melamine exposure is far below the established TDI values, additional work may be needed to clarify the risks of chronic, low-level exposure.

We hypothesized that meat would be an important contributor to melamine exposure because the contamination of animal feed is common [17,54,55,56] and melamine has been detected in animal tissues [13,14,15,57,58]. We did not observe significant associations with most meat types, but processed meats were positively associated with melamine exposure. This association was not statistically significant when using an adjusted threshold to reduce the risk of type I error. However, other studies provide support for an association of processed meats with melamine exposure. Shi et al. identified processed meats as a dietary source of melamine among adults in Shanghai [16]. In their analysis of foodstuffs in New York, Zhu et al. found variable melamine concentrations across meat and seafood items, but several processed items such as chicken hot dogs and turkey bacon had melamine concentrations similar to or higher than fresh meats [17]. The melamine contamination of food through migration from food packaging and/or processing materials [17,56] may lead to the contamination of processed meats, but additional study is necessary to improve the understanding of the sources and levels of melamine contamination in meats.

We observed a positive association between whole grains and melamine, consistent with other studies [16,17] and corroborated in our dietary pattern analysis. We found that a dietary pattern rich in whole grains and certain other plant-based foods such as fruits and soy was associated with melamine exposure. The widespread use of the pesticide cyromazine may lead to the melamine contamination of plant-based foods, as this pesticide is metabolized to form melamine through a dealkylation reaction in plants and animals [16]. Among 557 crops sampled in China, wheat had the highest level of melamine contamination, which was suspected by the authors to be related to cyromazine use [11]. Water and soil contamination, especially in close proximity to factories using or producing melamine, may also contribute to the contamination of crops destined for the human food supply [11,59].

Our study has several strengths, including the use of detailed dietary data from a 24-h recall. Because 90% of melamine is excreted through the urine within 24 h of consumption [32], a 24-h food recall is an ideal method for examining dietary sources of melamine. Additionally, the design of NHANES allowed us to generate nationally representative estimates of melamine exposure. To our knowledge, this is the first study to examine the association between dietary intake and melamine exposure in a representative sample of US children and adults. A primary limitation of this study is that the data were gathered in 2003–2004, which may not be reflective of the US population and food landscape today. Additionally, our analysis could not account for melamine exposure arising from food contact with melamine-containing tableware. Melamine migration from these materials is generally thought to be low [60], yet it could be an important source among children, and can be amplified by acidic and/or heated foods [1,60]. Furthermore, our study included data from relatively few children and did not include data from participants younger than 8 years old. Melamine exposure is widespread among children [18], and because of differing metabolisms, dietary choices, and exposure sources between children and adults [49], it will be important to further examine the associations of diet and melamine exposure across age groups in future studies.

## 5. Conclusions

In conclusion, this study suggests that processed meats, whole grains, and possibly other plant-based food items may be important sources of melamine exposure in the US diet. Further research, especially studies including young children, should be conducted to clarify the dietary sources of melamine in the current US food supply. The direct measurement of the melamine concentrations in foods would also improve our understanding of melamine exposure sources and inform approaches to reduce exposure. Additional research is needed to assess exposure to structural analogs of melamine, and to examine the potential health risks of chronic low-level exposure to melamine and related compounds.

## Figures and Tables

**Table 1:** Geometric means (SE) of urinary melamine, creatinine, creatinine-adjusted melamine, and estimated daily intake (EDI) for the US population and sociodemographic subgroups in National Health and Nutrition Examination Survey (NHANES) 2003–2004.

Population Group	*n*	Urinary Melamine (ng/mL)	Urinary Creatinine (mg/dL)	Creatinine-Adjusted Melamine (µg/g)	*p*-Value ^1^	EDI (µg/kg Body Weight)	*p*-Value ^1^
Total	478	1.325 (0.143)	142.4 (4.04)	11.563 (1.235)		0.251 (0.026)	
Sex					<0.05		0.21
Male	227	1.497 (0.224)	140.7 (7.71)	10.642 (1.825)		0.259 (0.044)	
Female	251	1.176 (0.179)	93.7 (5.14)	12.553 (1.759)		0.244 (0.034)	
Age Group					<0.05		<0.05
<18	124	0.689 (0.205)	119.8 (8.08)	5.125 (1.551)		0.119 (0.036)	
≥18 and <40	164	1.050 (0.214)	105.2 (4.57)	8.765 (1.845)		0.189 (0.040)	
≥40	190	1.958 (0.234)	75.0 (6.69)	18.617 (2.476)		0.397 (0.054)	
BMI Status ^2,3^					<0.001		<0.001
Underweight	10	0.746 (0.548)	113.1 (6.27)	8.888 (7.828)		0.184 (0.164)	
Normal/healthy	196	1.182 (0.235)	141.9 (7.83)	11.670 (2.503)		0.251 (0.054)	
Overweight	151	1.433 (0.254)	175.0 (48.73)	12.669 (2.629)		0.279 (0.057)	
Obese	115	1.489 (0.410)	165.3 (9.85)	10.489 (2.839)		0.227 (0.062)	
Poverty Income Ratio ^2^					0.26		0.22
<1.3	168	1.397 (0.196)	124.7 (7.64)	11.181 (1.442)		0.241 (0.032)	
≥1.3 and <1.85	66	1.232 (0.280)	131.8 (9.41)	9.238 (2.270)		0.201 (0.049)	
≥1.85 and <3.5	104	1.254 (0.315)	111.0 (10.22)	11.252 (2.949)		0.243 (0.062)	
≥3.5	120	1.353 (0.288)	104.5 (6.62)	12.955 (3.038)		0.284 (0.066)	
Race/Hispanic Origin					0.36		0.46
Mexican American	106	1.327 (0.371)	123.6 (4.29)	11.547 (2.984)		0.256 (0.066)	
Other Hispanic	20	0.643 (0.555)	108.6 (4.88)	5.201 (4.344)		0.112 (0.094)	
Non-Hispanic White	203	1.452 (0.185)	153.7 (12.21)	13.371 (1.807)		0.290 (0.039)	
Non-Hispanic Black	131	0.961 (0.154)	105.5 (27.48)	6.256 (1.277)		0.136 (0.028)	
Other including multiracial	18	2.165 (0.462)	149.0 (38.68)	20.525 (8.999)		0.452 (0.195)	
Physical Activity Level ^2,4^					<0.05		<0.05
Inactive	141	1.723 (0.308)	97.7 (3.79)	16.756 (2.912)		0.350 (0.060)	
Slightly Active	71	1.031 (0.345)	141.3 (16.18)	7.580 (2.843)		0.168 (0.062)	
Active	56	1.189 (0.360)	102.6 (20.16)	10.249 (3.616)		0.226 (0.080)	
Highly Active	30	1.273 (0.371)	105.9 (17.46)	11.377 (3.854)		0.258 (0.087)	
Smoking Status ^2,5^					0.44		0.35
Nonsmoker	288	1.383 (0.205)	102.7 (3.62)	13.466 (1.92)		0.292 (0.042)	
ETS-exposed	67	1.051 (0.315)	116.4 (16.45)	9.033 (2.983)		0.193 (0.063)	
Active Smoker	119	1.367 (0.259)	137.7 (5.58)	10.008 (1.971)		0.219 (0.043)	

^1^*p*-values for difference in creatinine-adjusted melamine and estimated daily intake (EDI) across levels of sociodemographic variables assessed using t-tests or ANOVA; ^2^ Descriptive statistics generated from combination of *n* = 10 imputed datasets with values imputed for missing BMI status (*n* = 6), PIR (*n* = 20), physical activity level (*n* = 180), and smoking status (*n* = 4); ^3^ Body mass index (BMI) status defined as underweight for children (age <20 years) at BMI percentiles <5 and adults (age ≥20 years) with BMI <18.5; normal weight for children at BMI percentiles ≥5 and <85 and adults with BMI ≥18.5 and <25; overweight for children at BMI percentiles ≥85 and <95 and adults with BMI ≥25 and <30; and obese for kids at BMI percentiles ≥95 and adults with BMI ≥30; ^4^ Physical activity level defined based on average daily time spent in moderate or vigorous activity bouts: inactive (≤15 min), slightly active (>15 and ≤30 min), active (>30 and ≤60 min), and highly active (>60 min); ^5^ Smoking status defined using serum cotinine values with <0.5 ng/mL defined as nonsmoker, ≥0.5 and ≤10 ng/mL defined as exposed to environmental tobacco smoke (ETS), and >10 ng/mL defined as active smoker.

**Table 2:** Expected change in creatinine-adjusted melamine concentration per one-unit increase in consumption of each food group (with 95% confidence intervals (CIs)) ^1^.

Food Group	Units	Minimally Adjusted Model ^2^		Fully Adjusted Model ^3^	
		Estimate (95% CI)	*p*-Value ^4^	Estimate (95% CI)	*p*-Value ^4^
Primary Analysis: Meats and Grains					
Total Meat, Poultry and Fish	ounces	−2.8% (−6.5, 1.1)	0.16	−3.2% (−7.3, 1.1)	0.14
Red Meat	ounces	−4.3% (−10.3, 2.1)	0.18	−5.3% (−11.3, 1.2)	0.11
Processed Meat	ounces	**9.7% (2.3, 17.7)**	**0.010**	**10.6% (2.7, 19.0)**	**0.007**
Poultry	ounces	−2.7% (−9.6, 4.7)	0.46	−2.5% (−9.6, 5.1)	0.50
Fish and Seafood (High ω-3)	ounces	9.3% (−4.8, 25.6)	0.21	8.3% (−9.2, 29.1)	0.38
Fish and Seafood (Low ω-3)	ounces	−2.2% (−19.8, 19.1)	0.82	−1.8% (−20.3, 21.1)	0.87
Total Grains	ounces	2.7% (−1.4, 6.9)	0.20	2.7% (−1.1, 6.6)	0.16
Whole Grains	ounces	**20.3% (6.2, 36.3)**	0.004 *	**17.4% (4.7, 31.7)**	0.006 *
Non-Whole Grains	ounces	1.7% (−2.6, 6.3)	0.44	1.9% (−2.1, 6.1)	0.36
Secondary Analysis: Other Main Food Groups					
Total Dairy	cups	−0.9% (−9, 7.9)	0.83	−2.0% (−10.1, 6.8)	0.64
Total Vegetables	cups	1.4% (−10.5, 14.9)	0.83	1.2% (−12.2, 16.6)	0.87
Total Fruits	cups	**11.8% (1.4, 23.3)**	**0.026**	11.9% (−1.9, 27.6)	0.09
Discretionary Oils	g	−0.3% (−1.1, 0.6)	0.53	−0.2% (−1.0, 0.5)	0.59
Discretionary Solid Fats	g	−0.2% (−0.9, 0.5)	0.63	−0.2% (−0.9, 0.5)	0.53
Added Sugars	teaspoons	−0.1% (−1.2, 1.0)	0.85	−0.2% (−1.3, 0.9)	0.73

^1^ Estimated from *n* = 10 imputations. Estimates derived from back-calculated regression coefficients in models predicting log-transformed creatinine-adjusted melamine; ^2^ Minimal models adjusted for creatinine (continuous), gender (male or female), and age (continuous); ^3^ Full models adjusted for creatinine (continuous), gender (male or female), age (continuous), Body mass index (BMI) status (underweight, normal/healthy weight, overweight, or obese), poverty index ratio (PIR) (continuous), race/ethnicity (Mexican American, other Hispanic, non-Hispanic White, non-Hispanic Black, or other including multiracial), smoking status (nonsmoker, exposed to environmental tobacco smoke (ETS), or active smoker), and physical activity level (≤15 min/day, >15 to ≤30 min/day, >30 to ≤60 min/day, or >60 min/day); ^4^ *p*-values bold if <0.05; * *p*-values marked with asterisks are significant at the Bonferroni-adjusted thresholds of 0.006 in the primary analysis and 0.008 in the secondary analysis.

**Table 3:** Factor loading matrix for dietary patterns of US adults and children in NHANES 2003–2004 ^1^.

Food Group	Western Pattern	Prudent Pattern	American-Healthy Pattern	Convenience Pattern
Whole Grains	0.010	0.175	0.262	0.108
Non-Whole Grains	0.267	−0.058	0.097	−0.028
Dark-Green Vegetables	0.003	0.257	0.040	−0.086
Orange Vegetables	0.000	0.254	0.002	−0.142
White Potatoes	0.066	0.066	−0.326	0.195
Other Starchy Vegetables	0.005	0.098	−0.068	0.027
Tomatoes	0.176	0.011	0.147	−0.322
Other Vegetables	0.107	0.263	−0.038	−0.327
Citrus, Melons, and Berries	0.033	0.108	0.176	0.013
Other Fruits	0.002	0.161	0.347	0.090
Milk	0.070	0.001	0.254	0.291
Yogurt	−0.007	0.060	0.176	0.038
Cheese	0.222	−0.138	0.164	−0.080
Red Meat	0.103	−0.007	−0.222	−0.261
Organ Meat	0.017	0.006	−0.030	0.036
Processed Meat	0.103	−0.111	0.048	0.146
Poultry	0.031	0.118	−0.148	0.119
Fish and Seafood (High ω-3)	−0.002	0.153	0.022	−0.046
Fish and Seafood (Low ω-3)	0.006	0.144	−0.018	−0.004
Eggs	0.063	0.012	−0.077	−0.038
Soy Products	0.000	0.140	0.153	−0.136
Nuts and Seeds	0.052	0.245	−0.055	0.276
Legumes	0.047	0.030	0.037	−0.138
Discretionary Oils	0.146	0.290	−0.203	0.259
Discretionary Solid Fats	0.300	−0.113	0.020	0.062
Added Sugars	0.196	−0.064	−0.126	0.190
Alcoholic Beverages	0.046	0.050	−0.237	−0.195

^1^ Factor loadings represent the magnitudes and directions of associations with factors (dietary patterns) and can range from −1.0 to 1.0. Stronger negative factors are shaded in red, stronger positive factors are shaded in green, and yellow and lighter shades are intermediate.

**Table 4:** Fact-estimated regression coefficients (with 95% CI) for relationship of empirical dietary patterns with creatinine-adjusted melamine exposure ^1^.

Dietary Pattern	Minimally Adjusted Model ^2^	Fully Adjusted Model ^3^
Western	0.02 (−0.17, 0.20)	0.01 (−0.18, 0.20)
Prudent	−0.02 (−0.13, 0.09)	−0.01 (−0.14, 0.12)
American-Healthy	**0.15 (0.05, 0.25)**	**0.15 (0.05, 0.24)**
Convenience	−0.02 (−0.17, 0.14)	−0.01 (−0.16, 0.13)

^1^ Coefficients estimated from *n* = 10 imputations and in bold if *p* < 0.05; ^2^ Minimal models adjusted for creatinine (continuous), gender (male or female), and age (continuous); ^3^ Full models adjusted for creatinine (continuous), gender (male or female), age (continuous), BMI status (underweight, normal/healthy weight, overweight, or obese), PIR (continuous), race/ethnicity (Mexican American, other Hispanic, non-Hispanic White, non-Hispanic Black, or other including multiracial), smoking status (nonsmoker, ETS-exposed, or active smoker), and physical activity level (≤15 min/day, >15 to ≤30 min/day, >30 to ≤60 min/day, or >60 min/day). Coefficients and their 95% CIs are bold if *p* < 0.05.
