# Erratum: Melough, M.M.; et al. Dietary Sources of Melamine Exposure among US Children and Adults in the National Health and Nutrition Examination Survey 2003–2004. *Nutrients* 2020, *12*, 3844

**DOI:** 10.3390/nu13030958

**Published:** 2021-03-16

**Authors:** Melissa M. Melough, Deborah Foster, Amanda M. Fretts, Sheela Sathyanarayana

**Affiliations:** 1 Department of Child Health, Behavior, and Development, Seattle Children’s Research Institute, Seattle, WA 98101, USA; sheela.sathyanarayana@seattlechildrens.org; 2 Department of Epidemiology, School of Public Health, University of Washington, Seattle, WA 98195, USA; dfoster4@uw.edu (D.F.); amfretts@uw.edu (A.M.F.); 3 Department of Pediatrics, University of Washington, Seattle, WA 98105, USA

The authors have requested that the following changes be made to their paper [1].

The original authors wish to add Dr. Amanda M. Fretts as a coauthor to this paper, as she was mistakenly left off in the original published version. Contributions by Dr. Fretts included study conceptualization, supervision, and revision/editing of the manuscript. She approved the final manuscript for publication during August 2020. Unfortunately, it was not recognized by the authors until after publication that Dr. Fretts had been mistakenly left off of the author list.

We would like to apologize for any inconvenience caused to the authors and readers by this mistake. The published version will be updated on the article webpage, with reference to this notice.
